# Parvalbumin expression changes with retinal ganglion cell degeneration

**DOI:** 10.3389/fnins.2023.1227116

**Published:** 2023-10-12

**Authors:** Yuan Liu, Rossana Cheng He, Gustavo C. Munguba, Richard K. Lee

**Affiliations:** ^1^Bascom Palmer Eye Institute, University of Miami Health System, Miami, FL, United States; ^2^Department of Surgery, Mount Sinai Hospital, New York, NY, United States; ^3^Envision Eye Specialists, Ocala, FL, United States

**Keywords:** retinal ganglion cell, glaucoma, optic nerve, microaaray, molecular marker, optic nerve crush

## Abstract

**Background:**

Glaucoma is one of the main causes of irreversible visual field loss and blindness worldwide. Vision loss in this multifactorial neurodegenerative disease results from progressive degeneration of retinal ganglion cells (RGCs) and their axons. Identifying molecular markers that can be measured objectively and quantitatively may provide essential insights into glaucoma diagnosis and enhance pathophysiology understanding.

**Methods:**

The chronic, progressive DBA/2J glaucomatous mouse model of glaucoma and C57BL6/J optic nerve crush (ONC) mouse model were used in this study. Changes in PVALB expression with RGC and optic nerve degeneration were assessed via gene expression microarray analysis, quantitative real-time polymerase chain reaction (qRT-PCR), Western blot and immunohistochemistry.

**Results:**

Microarray analysis of the retinal gene expression in the DBA/2J mice at different ages showed that the expression of PVALB was downregulated as the mice aged and developed glaucoma with retinal ganglion cell loss. Analysis of qRT-PCR results demonstrated PVALB at the mRNA level was reduced in the retinas and optic nerves of old DBA/2J mice and in those after ONC compared to baseline young DBA2/J mice. PVALB protein expression measured by Western blot was also significantly reduced signal in the retinas and optic nerves of old DBA/2J mice and those eyes with crushed nerves. Immunohistochemical staining results demonstrated that there were fewer PVALB-positive cells in the ganglion cell layer (GCL) of the retina and staining pattern changed in the optic nerve from old DBA/2J mice as well as in mice eyes following ONC.

**Conclusion:**

PVALB is abundantly expressed both by RGCs’ soma in the retinas and RGCs’ axons in the optic nerves of C57BL/6J. Furthermore, the expression level of PVALB decreases with RGC degeneration in the glaucomatous DBA/2J mice and after ONC injury of C57BL6/6J, indicating that PVALB is a reliable RGC molecular marker that can be used to study retinal and optic nerve degeneration.

## 1. Introduction

Glaucoma is the second leading cause of irreversible blindness globally (Quigley and Broman, 2006). It was estimated that 64.3 million people were affected by glaucoma in 2013 and the study predicted that the number would reach 76 million in 2020 and 111.8 million in 2040 (Tham et al., 2014). Glaucoma is a group of optic neuropathies characterized by the progressive death of RGCs and degeneration of the optic nerve, which result in visual field defects and blindness. RGCs, located in the innermost layer of the retina, function to integrate information from the photoreceptors via bipolar and amacrine cells and project into the brain for higher-order visual processing. RGC degeneration impairs information transmission in the optic pathway, rendering glaucoma a neurodegenerative disease.

Glaucoma is a multifactorial disease. A number of theories have been proposed for the pathogenesis and progression of glaucoma, which include intraocular pressure (IOP) elevation (Gordon et al., 2002; Heijl et al., 2002; Kass et al., 2002; Leske et al., 2007), neurotrophin deprivation (Pease et al., 2000; Quigley et al., 2000), vascular dysregulation (Hayreh, 1969; Chung et al., 1999; Hayreh et al., 2004), and neuroinflammation (Tezel, 2013; Kamat et al., 2016). Given the complex etiology of neurodegeneration in glaucoma, it is important to identify biomarkers that would allow for a better understanding of the disease, the monitoring of the disease progression, and the development of therapeutics.

PVALB is a calcium buffer that belongs to the large family of EF-hand calcium-binding proteins, which contain the classical EF-hand motif of helix–loop–helix that allows for calcium or magnesium binding (Kretsinger and Nockolds, 1973). Its calcium-binding capacity allows PVALB to regulate intracellular calcium concentration and thus, PVALB plays a role in various calcium-dependent signaling pathways (Schwaller, 2010). PVALB was first identified and characterized in carp muscle as a calcium-binding carp myogen (Konosu et al., 1965; Nockolds et al., 1972). Implicated in the process of muscle relaxation by binding and shuttling Ca^2+^ to the sarcoplasmic reticulum, PVALB’s expression was also found in the fast-twitch muscles of most mammals, including mice, horses, and humans (Heizmann et al., 1982). Additionally, PVALB is expressed throughout the central and peripheral nervous system, including the cerebellum, hypothalamus, hippocampus, olfactory bulb, retina, and the spinal ganglia (Heizmann, 1984; Celio, 1990). PVALB, along with other EF-hand calcium-binding proteins such as calbindin D28K and calretinin, have been used as neuronal markers in various studies because of their abundance and specificity in neurons, which allow for the differentiation and characterization neuron subpopulations (Baimbridge et al., 1992). As a result, the function of a particular subpopulation of neurons in a neural circuitry can be further investigated and its susceptibility to neurodegenerative diseases can be assessed.

PVALB is expressed in the retina of various vertebrates; however, its immunoreactivity pattern varies across different species (Sanna et al., 1993). In the mammalian retina of rabbits, rats, and mice, PVALB has consistently showed immunoreactivity in the inner nuclear layer (INL) and the GCL, labeling amacrine cells and RGCs (Wäussle et al., 1993; Casini et al., 1995; Haverkamp and Wässle, 2000). In the retina of adult C57BL/6J mice, it was estimated that 85.93% of PVALB-immunoreactive cells were RGCs and 28.98% of RGCs expressed PVALB (Kim and Jeon, 2006). Furthermore, these PVALB-immunoreactive RGCs showed heterogeneity in their morphology, labeling different subpopulations of RGCs (Kim and Jeon, 2006).

In this study, we aimed to determine if PVALB is a reliable RGC marker in adult mouse eyes and if PVALB expression can be used to track progressive loss of RGCs, such as glaucomatous DBA/2J mouse model and optic nerve injury model. The ONC injury causes axonal degeneration and RGC death (Barron et al., 1986; Li et al., 1999; Leung et al., 2008). This experimental disease model allows for the study of mechanisms underlying axonal injury-induced RGC death and survival. On the other hand, the DBA/2J mice have become a widely used animal model for the study of glaucoma due to its similarities with the human disease. The DBA/2J mouse strain is mouse model of hereditary glaucoma; these mice spontaneously and progressively develop a number of ocular abnormalities with age. Starting at approximately 6 months of age, the DBA/2J mice develop pigment dispersion, iris transillumination, iris atrophy, and anterior synechia (John et al., 1998). Elevation in intraocular pressure (IOP) is detected from 9 to 12 months. By 12 months of age, the majority of animals have significant RGC loss and optic nerve degeneration (Libby et al., 2005). Using these two different animal models, we examined the changes in the expression of PVALB with retinal degeneration at both the transcript and protein level. Immunofluorescence experiments confirm that PVALB is preferentially expressed in non-crushed C57BL6/J and young DBA/2J mouse. Genechip analysis confirm by RT-PCR show that the expression of PVALB decreased with age in the DBA/2J mice and following ONC. Western blot results further supported our conclusion.

## 2. Methods

### 2.1. Animals

C57BL/6J and DBA/2J mice were purchased from the Jackson Laboratory (Bar Harbor, ME). They were bred and handled according to the ARVO Statement for the Use of Animals in Ophthalmic and Vision Research. All animal procedures were approved by the University of Miami Institutional Animal Care and Use Committee.

### 2.2. GeneChip microarray

Total RNA from 4 to 6 pooled whole retinas of C57BL/6J and DBA/2J mice was isolated using TRIzol Reagent and Qiagen RNeasy column purification. Samples were sent to Expression Analysis, Inc. (Durham, NC) for gene expression profile service using the Affymetrix GeneChip Mouse Genome 430 2.0 Array (see Munguba et al., 2013 for more details).

### 2.3. Optic nerve crush

The ONC was performed according to the method used by Li and colleagues with slight modifications (Li et al., 1999). 3-month-old C57BL/6J mice were anesthetized by intraperitoneal injection with 0.005 ml of anesthesia per gram of body weight, containing ketamine (15 mg/ml) and xylazine (3 mg/ml). An incision was made to the conjunctiva to allow access to the posterior region of the globe. The optic nerve was exposed through a small window made through blunt dissection. At a site approximately 2 mm posterior to the globe, the optic nerve was clamped with forceps for 8 s. The contralateral eye was used as the uncrushed control. The animals were sacrificed 1 month after the ONC.

### 2.4. Reverse transcription and quantitative real-time PCR

Whole retinas were isolated from glaucomatous DBA/2J and age-matched control C57BL/6J mice as well as from 1-month post-ONC mice. Four pooled retinas from each group were homogenized on ice with a mortar and pestle (Pellet Pestle Cordless Motor Tissue Grinder and Kimble Kontes Pellet Pestle, Kimble Chase, Rockwood, TN) and total RNA was isolated using TRIzol Reagent (Lot #54101, Invitrogen, Carlsbad, CA). cDNA was synthesized from 2 μg of total RNA using Superscript III Reverse Transcriptase (Lot #1517623, Invitrogen) according to the manufacturer’s protocol. A 1:20 dilution was made to the resulting cDNA samples. All samples were stored at −20°C until qRT-PCR analysis.

The following primer pairs were designed using Primer-BLAST:

**Table 1:** 

Gene	Forward primer (5′–3′)	Reverse primer (5′–3′)
PVALB	TCTTTTCGCACTTGCTCTGCC	TCAGAATGGACCCCAGCTCATC
Thy1	CAAGGTCCTTACCCTAGCCAA	CCAGCTTGTCTCTATACACACTG
β-actin	CAACGGCTCCGGCATGTGC	CTCTTGCTCTGGGCCTCG

The following components were added to each reaction well for a total volume of 20 μl per qRT-PCR reaction: 10 μl of iQ SYBR Green Supermix (Lot #010171B, Bio-Rad, Hercules, CA), 7.2 μl of diethylpyrocarbonate (DEPC)-treated water, 0.4 μl (an equivalent of 0.04 nmol) of forward primer, 0.4 μl (an equivalent of 0.04 nmol) of reverse primer, and 2 μl of the 1:20 dilution of the cDNA template. Samples were run in quadruplicates using the CFX Connect Real-Time PCR Detection System (Bio-Rad) and all samples were analyzed together on a single 96-well plate with CFX Manager Software (Bio-Rad). The qRT-PCR was repeated 5 times. All primer sets were exposed to identical three-step cycling conditions: 94°C for 5 min (initial denaturation and enzyme activation), followed by 40 cycles of 94°C for 30 s (denaturation), 55°C for 30 s (annealing), 72°C for 40 s (extension). β-actin was used as an endogenous control to normalize the mRNA expression of parvalbumin and Thy1 in each sample. The transcript levels were quantified by the comparative quantification method – the double delta Ct method – using β-actin as the reference gene and the C57BL/6J as the calibrator.

### 2.5. Immunofluorescence and confocal microscopy

After transcardial perfusion with phosphate-buffered saline (PBS) and 4% paraformaldehyde, eyes were enucleated from the following mice: 2.3- and 20-month-old DBA/2J, 3- and 20-month old C57BL/6J mice, and 1-month post ONC mice. The globes were fixed in 4% paraformaldehyde. Then, the globes were embedded in paraffin, sectioned at 4 μm thickness, and mounted onto glass slides. The tissue sections underwent deparaffinization with xylene overnight and rehydration during which they were subjected to an ethanol gradient wash (100% ethanol, 95% ethanol, 75% ethanol, 50% ethanol, and dH_2_O; for 3 min each). To restore the immunoreactivity of the epitopes, the sections were steam-heated in 1× Rodent Decloaker (Biocare Medical, Concord, CA) for 40 min. Subsequently, the sections were blocked with Rodent Block M (Biocare Medical) for 20 min to reduce nonspecific antibody binding. Tissue slides were incubated for 2 nights with primary antibodies diluted in 1× PBS containing 0.5% Triton, washed with 1× PBS, incubated with secondary antibodies diluted in 1× PBS for 1 h, and washed with 1× PBS. Lastly, coverslips were mounted onto the slides using VECTASHIELD Antifade Mounting Medium with DAPI (Vector Laboratories, Burlingame, CA). Confocal microscopy images were taken using the Leica DM6000 B microscope (Leica Microsystems, Wetzlar, Germany) with a 40x oil immersion objective.

Primary antibodies used were: 1:200 rabbit polyclonal anti-parvalbumin antibody (Lot GR296273-1, Abcam, Cambridge, MA), 1:200 guinea pig polyclonal anti-RBPMS antibody (Lot NB916o, PhosphoSolutions, Aurora, CO), and 1:200 rat monoclonal anti-Thy1 antibody (Lot GR317580-1, Abcam). Secondary antibodies used were: 1:200 Cy3 donkey anti-rabbit IgG (Jackson ImmunoResearch, West Grove, PA), 1:200 Cy5 donkey anti guinea pig IgG (Jackson ImmunoResearch, West Grove, PA), and 1:200 Alexa Fluor 488 donkey anti-rat IgG (Jackson ImmunoResearch, West Grove, PA).

### 2.6. Western blots

Retinas from 4 mice in each group were homogenized and digested with RIPA buffer. Total protein extract (25 μg) was loaded on a 12% polyacrylamide gel. After electrophoresis, proteins were transferred onto polyvinyl difluoride (PVDF) membranes (ThermoFisher, Catalog number: 88518). Nonspecific bindings sites were blocked by immersing the membrane in 5% milk in Tris-buffered saline Tween (TBST) for 1 h at room temperature. Membranes were incubated with primary antibody overnight, washed 3 times with TBST for 15 min, incubated with secondary antibody for 1 h at room temperature, washed 3 times with TBST for 15 min and exposed with ECL (ThermoFisher, Catalog number: 32106). To quantify, the membranes were scanned by gel scanner using software.

Primary antibodies used were: 1:1000 rabbit polyclonal anti-parvalbumin antibody (Lot GR296273-1, Abcam, Cambridge, MA), 1:1000 mouse monoclonal anti-parvalbumin antibody (ab8245, Abcam, Cambridge, MA). Secondary antibodies used were 1:2000 HRP donkey anti-mouse IgG (Jackson ImmunoResearch, West Grove, PA), Secondary antibodies used were 1:2000 HRP donkey anti-rabbit IgG (Jackson ImmunoResearch, West Grove, PA).

### 2.7. Statistical analysis

Each animal was used in a single experiment and entered as an individual data point. Statistical analyses were performed using GraphPad Prism software (GraphPad Software, Inc., La Jolla, CA). Paired student *t*-test was used to compare two groups. *p* values less than 0.05 were considered statistically significant. Multiple comparison analyses were conducted using Analysis of Variance (ANOVA) with Tukey’s *post hoc* test. *p* values less than 0.05 were considered statistically significant.

## 3. Results

### 3.1. Parvalbumin expression is reduced in mouse retina after optic nerve crush

**Figure 1:**
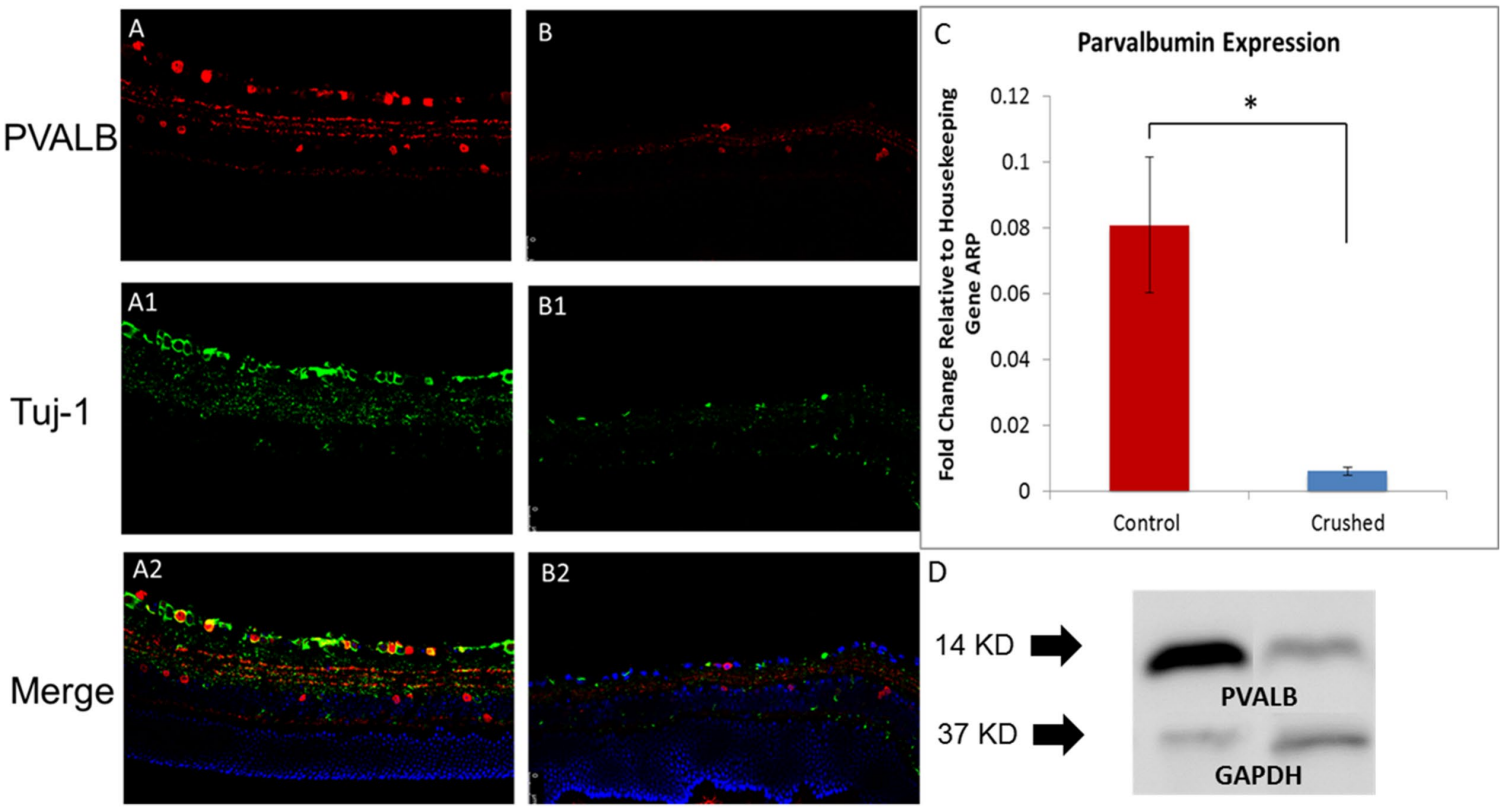
PVALB expression decreased in the retinas from mice that have been crushed. **(A)** PVALB immunohistochemistry (red) and its colocalization with neuronal markers, Tuj1(green), in a 3-month-old C57BL/6J mouse retina. PVALB mainly showed immunoreactivity in the NFL, GCL, and IPL. **(B)** Both PVALB and Tuj1 signal decreased after crush. **(C)** PVALB mRNA expression levels decreased 1 month after ONC by qRT-PCR. Using β-actin as the internal control, the expression levels of PVALB were reduced to 21% of the uncrushed control (*p < 0.05, student *t* test, *n* = 10 for each group). **(D)** Western blot results demonstrated decreased expression of PVALB in retinas from crushed mice. Left lane, retina extracts from mice have been crush for 4 weeks. Right lane, retina extracts from age-matched non-crushed mice. Bottom lane, GAPDH staining used as loading control (*n* = 4 for each group).

In the 3-month-old C57BL/6J retina, PVALB demonstrated abundant expression in the GCL and NFL (Figure 1A) retinal layers. PVALB was also immunoreactive in the inner plexiform layer (IPL), thereby showing a three-band stratification pattern in the retina. Additionally, PVALB labeled a number of cells in the proximal inner nuclear layer. Antibodies specific for Tuj-1, a protein commonly used as a neuron marker (Barnstable and Dräger, 1984; Rodriguez et al., 2014), was used in conjunction with the antibody against PVALB in the immunohistochemical staining to further characterize the staining pattern of PVALB (Figures 1A,B). In the NFL, the majority of PVALB staining colocalized with Tuj-1 (Figure 1A). In the GCL, PVALB-positive cells showed colocalization with Tuj-1 (Figure 1A). In the IPL, PVALB labels showed very good lamination along this retinal layer. In the mice that underwent unilateral ONC, the crushed eye (Figure 1B) had fewer PVALB-positive cells in comparison to the contralateral, uncrushed control 1 month after the crush (Figure 1A).

To further examine the changes in the expression of PVALB with RGC degeneration, qRT-PCR and Western blot were performed. The qRT-PCR results showed that 4 weeks after the ONC injury, the transcript levels of PVALB in the crushed eyes significantly decreased in comparison to the uncrushed control. The level of PVALB expression was reduced to 21% of the control level (Figure 1C). The level of Thy1 expression, a recognized RGC marker, was also reduced to 30% of the control. When PVALB expression level was normalized to Thy1, no significant expression differences were observed between crushed and non-crushed control, indicating strong correlation with Thy1 and PVALB in RGCs. The protein expression of PVALB was significantly reduced in retinas from crushed eyes compared to the non-crushed contralateral eyes (Figure 1D) as demonstrated by Western blotting.

### 3.2. Parvalbumin expression change in mouse optic nerve after crush

**Figure 2:**
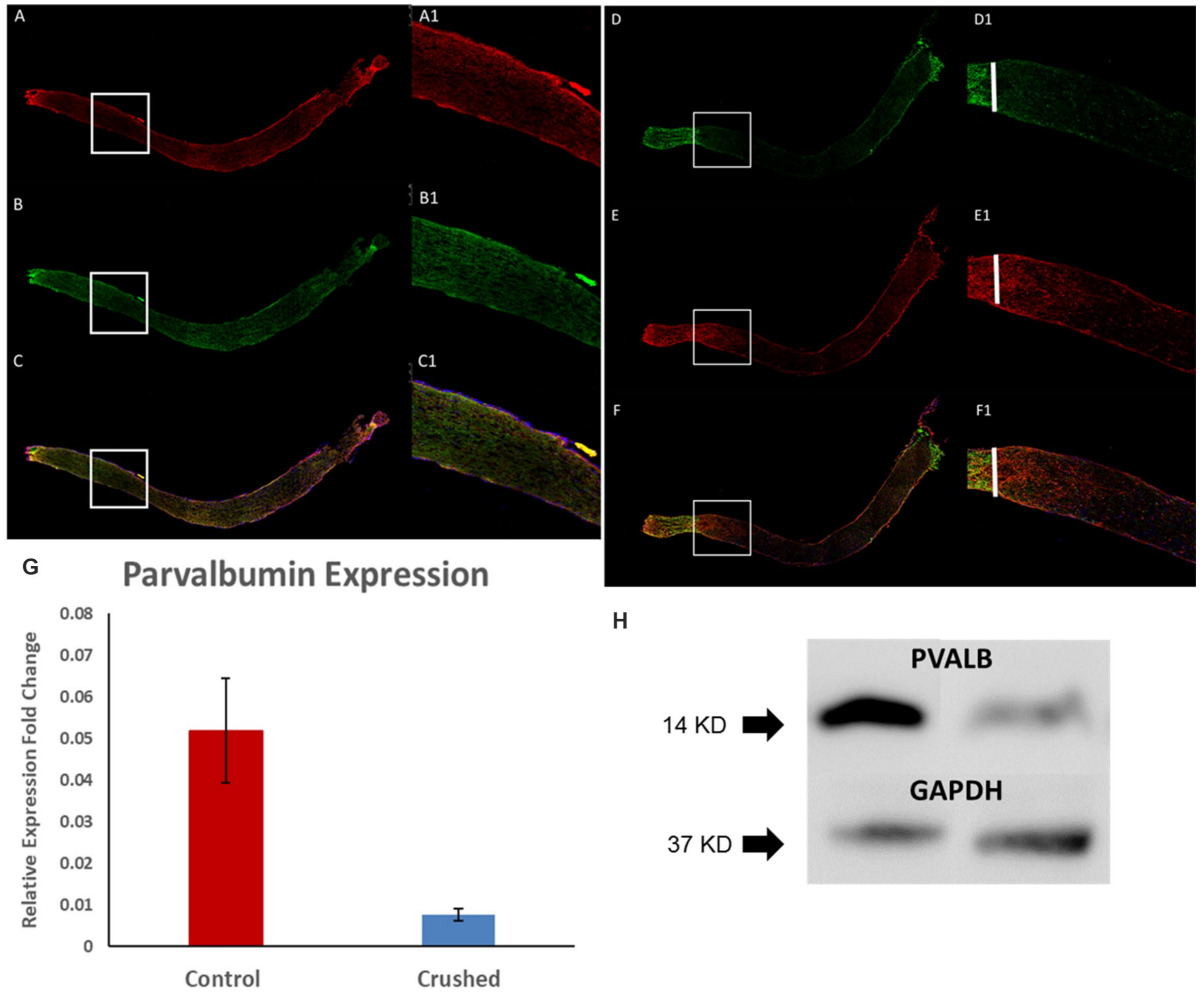
PVALB expression decreased in the optic nerves from mice that have been crushed. **(A–C)** PVALB immunohistochemistry (red) and its colocalization with neuronal fiber markers, Tuj1(green), in a 3-month-old C57BL/6J mouse optic nerve. **(D–F)** Both PVALB and Tuj1 signal decreased after crush. We can hardly observe PVALB and Tuj-1 signal after crush site. White bar labels crushed site. **(A1–F1)** are the amplified images from the square site. **(G)** PVALB mRNA expression levels decreased 1 month after ONC by qRT-PCR. Using β-actin as the internal control, the expression levels of PVALB were reduced to 23% of the uncrushed control (*p < 0.05, student *t* test, *n* = 10 for each group). **(H)** Western blot results demonstrated decreased expression of PVALB in optic nerves from crushed mice. Left lane, optic nerve extracts from mice have been crush for 4 weeks. Right lane, optic nerve extracts from age-matched non-crushed mice. Bottom lane, GAPDH staining used as loading control (*n* = 4 for each group).

In the 3-month-old C57BL/6J mice, we can observe positive PVALB staining in optic nerves and it colocalize with Tuj1 staining, which is a widely-used marker of nerve fibers (Figures 2A–C). After crush, nerve fibers underwent degeneration with diminished signal of both Tuj1 and PVALB (Figures 2D–F). The reduced expression of PVALB in optic nerves after crush was further verified by real-time PCR and Western blotting. Real time PCR show that the relative gene expression levels of PVALB significantly decreased at 4 weeks after crush compared to non-crushed controls (Figure 2G). Based upon western blot analysis, the amount of PVALB protein expression was significantly reduced in the optic nerves from crushed mice compared to non-crushed controls (Figure 2H).

### 3.3. Mouse glaucoma model shows reduced expression of parvalbumin in retina

**Figure 3:**
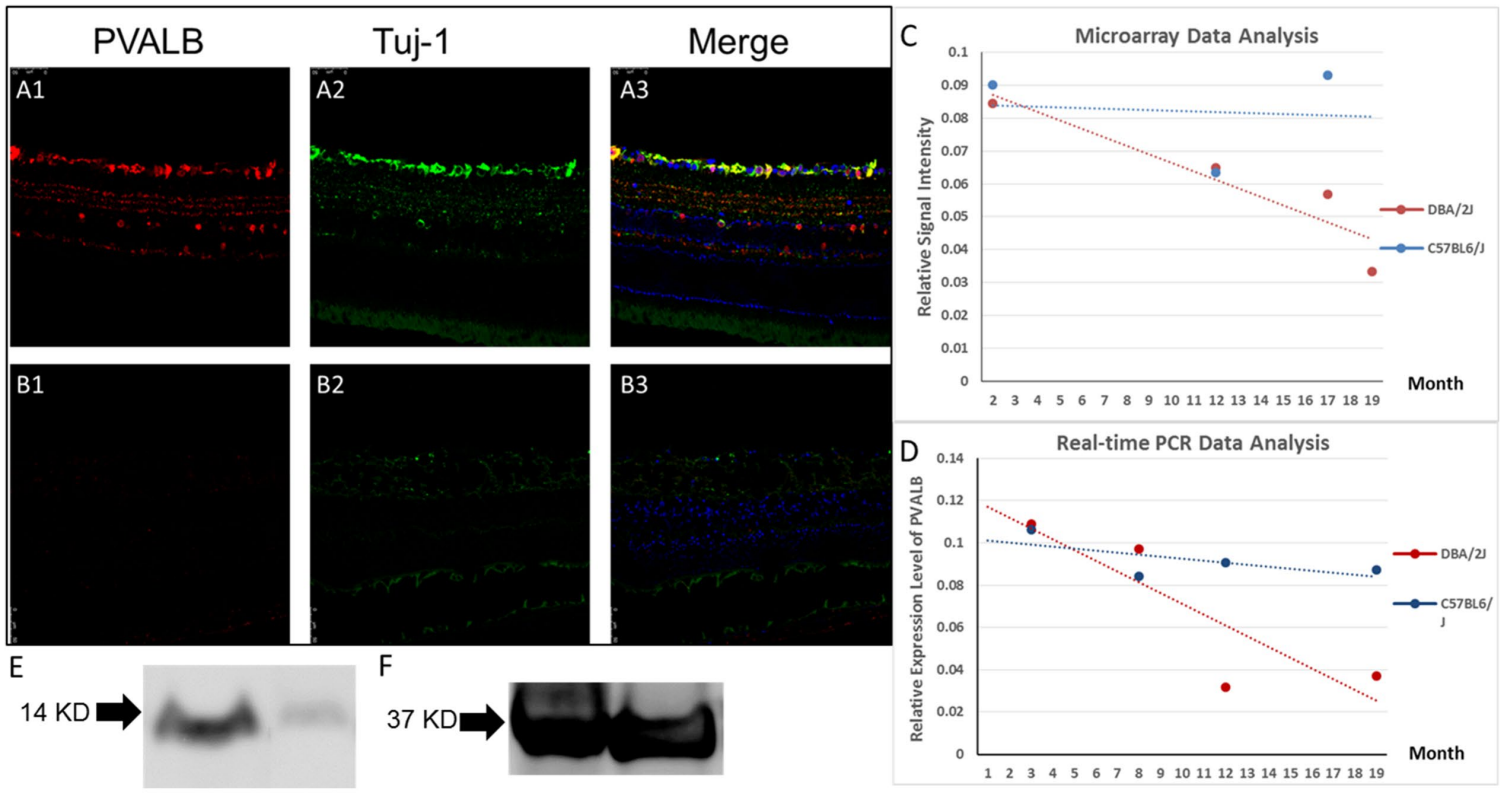
PVALB expression decreased in the retinas from aged DBA/2J mice. **(A1–A3)** PVALB immunohistochemistry (red) and its colocalization with neuronal markers, Tuj1 (green), in a 14-month-old C57BL/6J mouse retina. PVALB mainly showed immunoreactivity in the NFL, GCL, and IPL. **(B1–B3)** Both PVALB and Tuj1 signal decreased in 14-month-old DBA/2J mice. **(C,D)** Gene expression levels were measured from pooled whole retinas of the DBA/2J mouse model of glaucoma at different ages using the GeneChip Mouse Genome 430 2.0 Array. Signal levels of all Affymetrix probes for PVALB were averaged and plotted. Expression levels of PVALB were reduced as the DBA/2J mice aged **(C)**, while expression levels of PVALB in C57BL6/J mice did not have this decreasing trend. **(E)** Relative expression level of PVALB in C57BL6/J mice did not show decreasing trend. **(F)** PVALB mRNA expression levels decreased in aged DBA/2J mice quantified by qRT-PCR. Using β-actin as the internal control. **(G)** Western blot results demonstrated decreased expression of PVALB in retinas in aged DBA/2J mice. Left lane, retina extracts from 14-month-old DBA/2J mice. Right lane, retina extracts from age-matched C57BL6/J mice. Bottom lane, GAPDH staining used as loading control.

PVALB expression in DBA/2J mice eyes changed as they aged. Fewer RGCs showed immunoreactivity for PVALB in the glaucomatous, 14-month-old DBA/2J mouse retina compared to age-matched C57BL6/J mice and young 4-month old DBA/2J mice (Figures 3A1-3,3B1-3). Compared to age matched C57BL6/J mice and young DBA/2J mice, a significantly diminished ganglion cell layer and inter plexiform layer was barely observed in the aged DBA/2J mice. The lamination of cells labeled by PVALB also disappeared.

A further analysis of the microarray data comparing the transcriptional profiles of pooled whole retinas from DBA/2J and C57BL6/J mice at different ages revealed PVALB to be a potential RGC marker. The signal level of PVALB from the Affymetrix microarrays decreased as the mice aged: the averaged relative signal intensity of PVALB in the young DBA/2J mice was significantly higher than that in the old mice (Figure 3C). The relative signal intensity of PVALB in C57BL6/J did not have any obvious trend decrease (Figure 3D).

To further examine the changes in the expression of PVALB with RGC degeneration, qRT-PCR was performed. No significant differences between the transcript levels of PVALB in DBA/2J mouse retinas at the age of 3 and 8 months was observed (Figure 3F, *p* = 0.44, *n* = 20; unpaired *t*-test). However, as the mice aged and developed glaucoma, the PVALB transcript levels showed a reduction from 8-month-old to 12-month-old mice (Figure 3F; *p* < 0.0001, *n* = 20; unpaired *t*-test). Although the expression level showed an increase at the age of 19 months, it was still significantly lower than that at 8 months of age (Figure 3F, *p* = < 0.0001, *n* = 20; unpaired *t*-test). We did not observe similar expression changes in the C57BL6/J mouse line (Figure 3E).

The expression of Thy1, a cell-surface glycoprotein commonly used as a RGC marker (Barnstable and Dräger, 1984), was also examined in this study. We found that the transcript levels of Thy1 in the DBA/2J mouse retinas at different ages showed a similar trend compared to PVALB, with a decrease from the age 8 to 12 months, followed by a slight increase from 12 to 19 months. The correlation analysis showed that the PVALB and Thy1 is strongly correlated (r = 0.84, *p* = 0.051).

Furthermore, Western blot was used to analyze the expression of PV at the protein level in the mouse retina. PVALB protein expression level in 14 months DBA/2J mice and age-matched C57BL/6J mice was quantified by Western blotting. We detected almost complete loss of PVALB protein signal in 14 months DBA/2J mice retina based on the Western blotting results (Figure 3G).

### 3.4. Effect of elevated pressure on the amount of parvalbumin in mouse optic nerve

**Figure 4:**
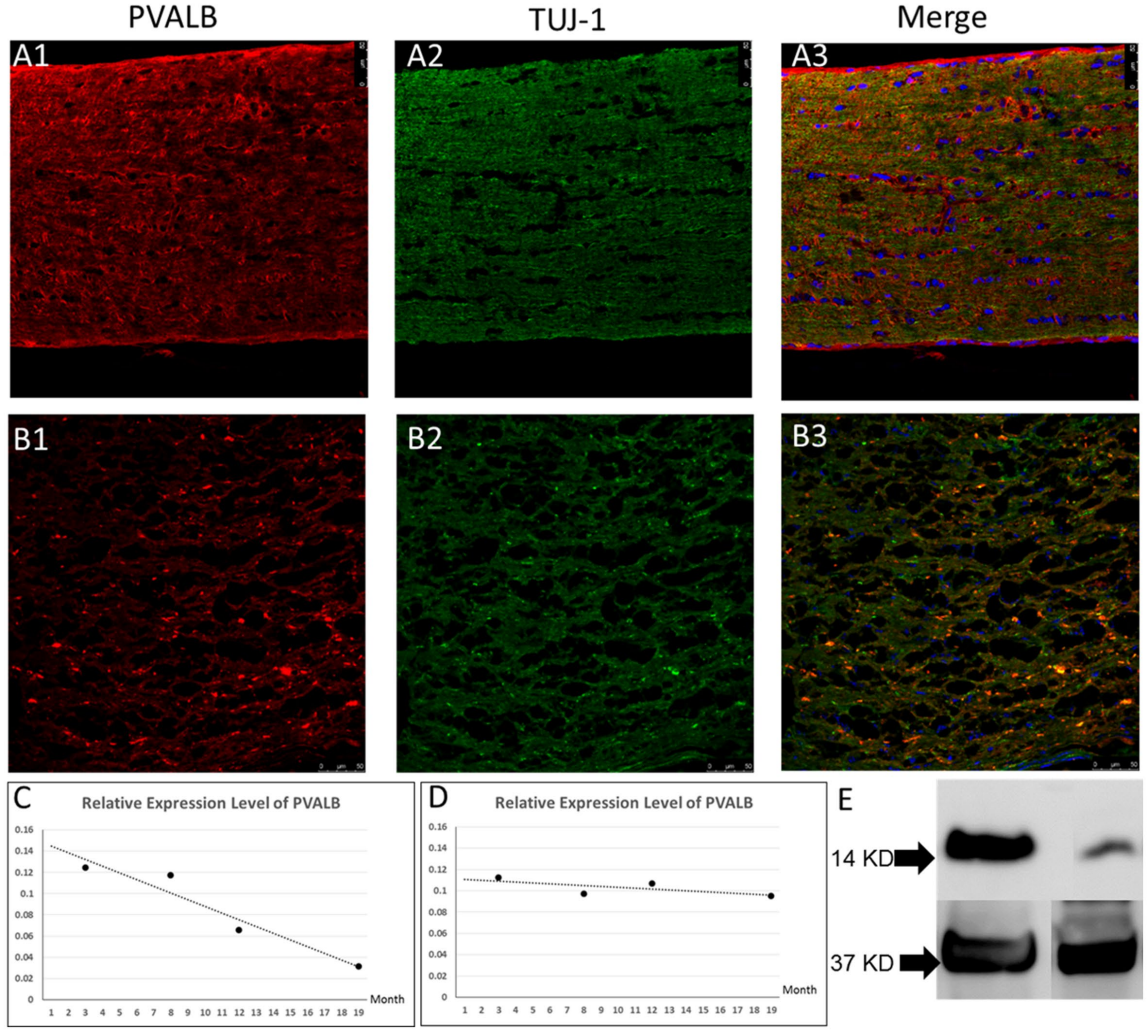
PVALB expression decreased in the optic nerves from aged DBA/2J mice. **(A1–A3)** Immunostaining of optic nerves from 4-month-old DBA/2J mouse. **(B1–B3)** Immunostaining of optic nerves from 14-month old DBA/2J mice. PVALB (red) and Tuj1 (green) were shown in the figures. We can observe immunostaining positive deposits in these optic nerves. **(C)** PVALB mRNA expression levels decreased in aged DBA/2J mice quantified by qRT-PCR. Using β-actin as the internal control. **(D)** Relative expression level of PVALB in C57BL6/J mice did not show decreasing trend. **(E)** Western blot results demonstrated decreased expression of PVALB in optic nerves from 14-month-old DBA/2J mice. Left lane, retina extracts from 14-month-old DBA/2J mice. Right lane, retina extracts from age-matched C57BL6/J mice. Bottom lane, GAPDH staining used as loading control.

Compared to control C57BL/6J mice optic nerve and young 4-month-old DBA/2J mouse, 14 months DBA/2J mice optic nerve demonstrated abnormal PVALB immunostaining pattern (Figures 4A1-3,4B1-3). We can find immunostaining positive deposits in the aged DBA/2J mice which were not seen in the wide type control and young DBA/2J mice. We quantified PVALB expression level in optic nerves by real-time PCR and Western blotting. Real-time PCR results demonstrated that the expression of PVALB sharply decreased at the age of 3.5 month, coinciding with the onset of glaucomatous retinal ganglion cell loss in the DBA/2J mouse line (Figure 4C). However, we did not observe same decreased expression of PVALB in C57BL6/J mice (Figure 4D). We also preformed western blot to detect PVALB protein expression change in the DBA/2J glaucomatous mouse line. The western blotting results demonstrated significantly reduced expression of PVALB in optic nerves of aged DBA/2J mice compared to age-matched BL6 control mice (Figure 4E).

## 4. Discussion

Glaucoma is a group of progressive optic neuropathies characterized by the degeneration of retinal ganglion cells (RGCs) and their axons, resulting in visual field loss. Early detection and timely intervention are crucial to prevent irreversible vision loss in glaucoma patients. Molecular markers offer valuable tools for improving the accuracy and efficiency of glaucoma diagnosis. Thus, identification of a reliable RGC marker to track disease progression is very important and a major focus of clinical research.

By utilizing molecular markers, clinicians can enhance the accuracy of glaucoma detection, monitor disease progression, and personalize treatment strategies. In the present study, we have identified PVALB as an effective marker with which to track RGC loss in both glaucoma animal model of disease and wide-used optic nerve injury model. We have demonstrated that PVALB is expressed by RGCs in both the C57BL/6J and DBA/2J mouse strains. Our findings are consistent with previous works, which showed that at least 8 different types of RGC in the mouse retina expressed PVALB (Kim and Jeon, 2006; Yi et al., 2012).

More importantly, we have shown that the expression of PVALB decreased with RGC degeneration in the mouse retina and optic nerves. Previous studies have found that the expression of PVALB was decreased after the induction of retinal ischemia/reperfusion in rats (Kim et al., 2010), ONC in rats (Hong et al., 2016), and chronic elevation of IOP in mice (Gunn et al., 2011). Similarly, our results showed that PVALB expression was reduced in the mouse retina and optic nerves 1 month after ONC, an injury model that results in 46% of RGC loss 30 days after crush (Templeton et al., 2009). The expression change of PVALB is correlated with Thy1, a well-recognized RGC markers, indicating PVALB expression level may also serves as a marker for RGC degeneration.

Changes in PVALB expression have not been previously studied in the DBA/2J mouse genetic model of glaucoma. Here, we showed that the expression of PVALB decreased as the age of the DBA/2J mice increased. DBA/2J mice develop age-dependent progressive ocular abnormalities including elevated IOP, iris atrophy and secondary angle closure. Anterior chamber pathology in DBA/2J mice typically has its onset at approximately 6 months of age, followed by elevation of IOP between 8 and 9 months, and severe optic nerve damage and RGC loss by 12 months of age in the majority of animals. Our microarray data and real-time PCR results demonstrated these pathological changes. No significant difference was observed between PVALB expression level at 3 months and 8 months. However, after 8 months, the PVALB expression level sharply decreased and kept decreasing as mice age increased with progressive retinal ganglion cell loss. The slight increase in the expression level of PVALB and Thy1 in the 19-month-old DBA/2J mice seen in the qRT-PCR data may be due to the variable and asymmetric disease progression within and between animals in this model (John et al., 1998; Schlamp et al., 2006).

Calcium signaling underlies numerous, vital cellular processes including proliferation, metabolism, muscle contraction, and neurotransmission (Brini et al., 2013). On the other hand, excessive intracellular Ca^2+^ can lead to cytotoxicity, triggering signaling cascades that result in cell death (Orrenius et al., 2003). Therefore, calcium-binding proteins are thought to play a protective role against neurodegeneration. Studies have demonstrated that the overexpression of PVALB offered protection to motor neurons against degeneration in amyotrophic lateral sclerosis (ALS; Elliott and Snider, 1995; Van Den Bosch et al., 2002). However, Laslo and colleagues found expression of parvalbumin within vulnerable motoneurons, suggesting that PVALB was not a reliable marker for resistance to degeneration in ALS (Laslo et al., 2000). The physiological function of PVALB in the retina is largely unclear and its potential neuroprotective role in retinal degeneration remains to be elucidated in future studies.

We proposed that PVALB may play a role in modulating calcium signaling in RGCs. Calcium signaling is crucial for neuronal function and survival, but excessive or dysregulated calcium levels can lead to cell damage or death. PVALB, as a calcium-binding protein, may help regulate calcium levels within RGCs and prevent calcium overload, which could be detrimental to their survival. Therefore, alterations in PVALB expression or function could disrupt calcium homeostasis and contribute to RGC degeneration.

Our study demonstrated that PVALB is abundantly expressed by RGCs’ soma in the mouse retina and RGCs’ axons in the optic nerve. Furthermore, the loss of RGCs seen in the transgenic DBA/2J mouse model of glaucoma as well as that induced by ONC injury was reliably identified by PVALB expression changes, demonstrating that PVALB may be used as a reliable RGC marker in different animal models of RGC degeneration to study the pathogenesis of various optic neuropathies.

## Data availability statement

The datasets presented in this study can be found in online repositories. The names of the repository/repositories and accession number(s) can be found in the article/supplementary material.

## Ethics statement

The animal study was reviewed and approved by the Institutional Animal Care and Use Committee, University of Miami.

## Author contributions

YL and RL designed the research. YL, RC, and GM performed the research. YL and RC analyzed the data. YL and RL wrote the manuscript. All authors contributed to the article and approved the submitted version.

## Funding

The Bascom Palmer Eye Institute is supported by NIH Center Core Grant P30EY014801 and a Research to Prevent Blindness Unrestricted Grant (GR004596-1). RL is supported by the Walter G. Ross Foundation. This work was partly supported by the Guitierrez Family Research Fund and the Camiener Foundation Glaucoma Research Fund.

## Conflict of interest

The authors declare that the research was conducted in the absence of any commercial or financial relationships that could be construed as a potential conflict of interest.

## Publisher’s note

All claims expressed in this article are solely those of the authors and do not necessarily represent those of their affiliated organizations, or those of the publisher, the editors and the reviewers. Any product that may be evaluated in this article, or claim that may be made by its manufacturer, is not guaranteed or endorsed by the publisher.
